# Effect of Film-Cooling-Hole Inclination on the Creep Performance of IN-738 Specimens

**DOI:** 10.3390/ma18081737

**Published:** 2025-04-10

**Authors:** Hao Yang, Qin Zhang, Jinke Lv, Han Li, Tiange Chu, Shaoyang Chen, Ke Wang

**Affiliations:** 1 School of Mechanics and Safety Engineering, Zhengzhou University, Zhengzhou 450001, China; yh1178790100@gs.zzu.edu.cn (H.Y.); 15670312831@163.com (Q.Z.); lvjinke1314@gs.zzu.edu.cn (J.L.); lihzzujx@gs.zzu.edu.cn (H.L.); chutiange@gs.zzu.edu.cn (T.C.); chensy0301@gs.zzu.edu.cn (S.C.); 2 Key Laboratory of Process Heat Transfer and Energy Saving of Henan Province, Zhengzhou University, Zhengzhou 450001, China

**Keywords:** film-cooling holes, inclination angle, creep lifetime, K-R damage model, life prediction

## Abstract

To investigate the effect of film-cooling-hole inclination on the creep performance of nickel-based superalloy IN-738 specimens, this study designed samples with film-cooling holes at four inclination angles: 0°, 30°, 45°, and 60°. High-temperature creep tests were conducted, and the fracture morphologies of the failed specimens were analyzed using scanning electron microscopy. The results indicate that under conditions of 800 °C and 350 MPa, the inclination angle of the film-cooling holes significantly influences the creep performance of the specimens, with creep lifetimes ranking in descending order as 0° > 60° > 45° > 30°. A fracture analysis revealed that creep failure in specimens with film-cooling holes primarily resulted from stress concentration at the hole edges, where cracks and voids frequently initiated. The creep fractures exhibited dimple-type failure characteristics localized around the film-cooling holes due to stress concentration. Simulations based on the K-R damage model were performed for the four different inclination angles, confirming the existence of stress concentration around the film-cooling holes. The numerical analysis results closely matched the experimental data. Furthermore, the node stress method was used to predict the creep rupture life of specimens with film-cooling holes, demonstrating high accuracy in life prediction.

## 1. Introduction

The gas turbine, often referred to as the “crown jewel” of industry, encompasses a wide range of applications, including various types of aerojet engines and their derivative gas turbines, heavy-duty (power generation) gas turbines, vehicular and industrial drive gas turbines, marine propulsion gas turbines, and various micro-gas turbines [1]. Among its components, turbine blades are considered the most critical parts due to their operation in the highest temperature, most complex stress, and harshest environmental conditions. The performance of turbine blades directly reflects the technological advancement of an engine model [2]. As the turbine inlet temperature continues to rise, the temperature resistance of superalloys has become insufficient to meet practical application requirements. Therefore, effective cooling methods are essential to reduce the wall temperature of turbine blades. Currently, machining film-cooling holes into turbine blades is one of the most effective techniques [3,4]. However, the presence of film-cooling holes disrupts the geometric integrity of the blade structure, resulting in stress concentration around the holes and making the blade material prone to creep failure under high temperatures [5,6,7]. Furthermore, due to the complex geometric shape of blades, some film-cooling holes are not perpendicular to the blade surface, making the inclination of the holes a critical factor affecting blade strength and lifespan.

Currently, domestic and international research on the mechanical performance of film-cooling holes primarily focuses on aspects such as hole arrangement and shape, while studies specifically addressing the effect of inclination angles are relatively limited. Ai Xing et al. [8] conducted creep tests on specimens with and without film-cooling holes and found that the fracture life of specimens without film-cooling holes was twice that of those with holes, indicating that film-cooling holes significantly reduce the creep performance. Zhang Dongxu et al. [9] designed flat plate specimens with three different inclination angles to study the effect of film-cooling-hole inclination on the fatigue performance of single-crystal alloys through high-temperature fatigue testing, concluding that inclination angles have a significant impact on fatigue performance. Zhang Dongxu et al. [10,11,12] also investigated the creep fracture mechanism of single-crystal superalloys, demonstrating that the presence of film-cooling holes reduces both the creep life and deformation capability of the specimens. Wang Xinmei et al. [13] examined the influence of hole shape on the high-temperature rupture life of single-crystal alloy cooling-blade specimens. They found that specimens with circular holes had a rupture life 1.3 times longer than those with expanded holes, and expanded-hole specimens in turn had a rupture life 1.3 times longer than those with W-shaped holes. Zhao Shicheng et al. [14] conducted creep tests on specimens with cylindrical, bucket-shaped, and dovetail-shaped film-cooling holes, finding that the average creep life of cylindrical and bucket-shaped holes was similar, and both were 17.4% and 15.8% longer than that of dovetail-shaped holes, respectively. Sun et al. [15] investigated the effect of multi-row film-cooling-hole structures on the creep performance of thin-walled specimens. Experimental results showed that specimens with single-row and double-row film-cooling holes had longer lifespans than those without holes; however, as the number of rows increased, the creep fracture life decreased. Liang et al. [16] used numerical methods to study the effect of different inclination angles on the creep performance of nickel-based single-crystal specimens. The results indicated that specimens with 30° inclined film-cooling holes exhibited the longest creep life, and the inclination angle significantly influenced the distribution of creep damage. Xiao Quanfen et al. [5], based on crystal plasticity theory, experimental results, and simulations at 980 °C and 250 MPa, found that the drilling direction significantly altered the stress distribution and the orientation of the stress axis, thereby effectively influencing the creep behavior and deformation mechanisms of nickel-based superalloys.

Regarding the life prediction of creep specimens with film-cooling holes, Webster G.A. and Hayhurst D.R. [17] identified a specific point in the minimum cross-section of notched round bar specimens where the stress state remains invariant under different stress exponents substituted into the Norton equation. This point is referred to as the “node”. The node stress method is now widely used to predict the creep life of specimens with notches or film-cooling holes. Its core concept [18] involves locating the node position under a multiaxial stress state and using the stress value at this point, combined with uniaxial creep test results, to predict the creep life under multiaxial conditions. Wen et al. [19,20,21,22], incorporating crystal plasticity theory and an improved node stress method, conducted extensive research on the mechanical performance of film-cooling holes. They proposed methods for predicting the creep and fatigue life as well as equivalent modeling methods for film-cooling holes. However, most of these studies focused on straight holes and did not fully address inclined holes commonly used in real turbine blade structures.

In this study, the nickel-based superalloy IN-738, commonly used in gas turbine blades, was selected as the material to investigate the high-temperature creep performance of specimens with film-cooling holes at four different inclination angles. Under conditions of 800 °C and 350 MPa, the effect of film-cooling-hole inclination on the creep performance of the specimens was systematically explored. After the tests, scanning electron microscopy (SEM) was employed to observe and analyze the fracture surfaces of the specimens. Finite element simulations based on the K-R damage model were conducted to study the distribution characteristics of strain, stress, and creep damage, as well as their relationship with the fracture cracks of the specimens. Finally, the node stress method was applied to predict the creep rupture life of the specimens.

## 2. Materials and Methods

### 2.1. Experimental Materials

The material used for the creep tests was the nickel-based superalloy IN-738, with its chemical composition listed in Table 1. The specimens underwent standard heat treatment procedures to ensure a uniform microstructure and eliminate residual stress. The heat treatment process was as follows: solution treatment at 1120 °C ± 10 °C for 2 h followed by air cooling and aging at 850 °C ± 10 °C for 24 h followed by air cooling.

### 2.2. Sample Preparation

**Figure 1:**
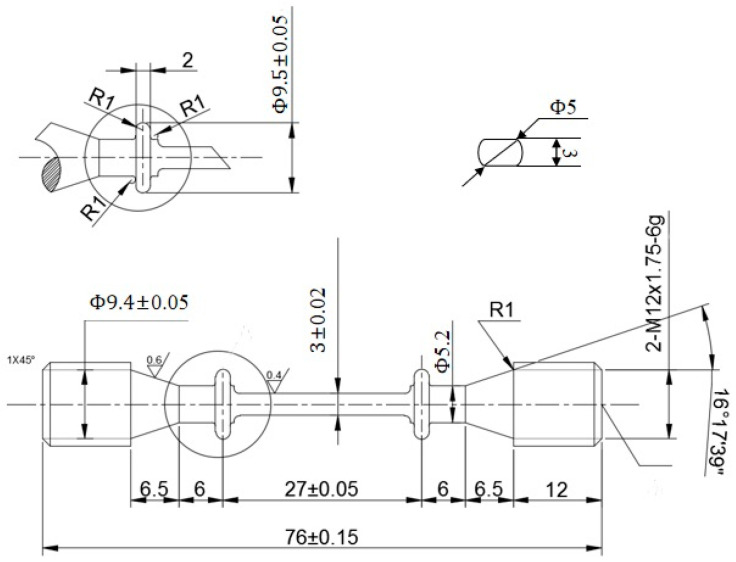
Dimensional diagram of a waisted cylindrical specimen.

Based on the structural characteristics of cooled blades and to facilitate subsequent drilling, the gauge section of the cylindrical specimen was modified to study the effect of different film-cooling hole structures on the creep performance of the specimens. The dimensions of the designed waist-shaped cylindrical specimen are shown in Figure 1. The total length of the specimen is 76 mm, with a gauge length of 27 mm. The cross-sectional schematic of the gauge section is also illustrated in the figure, with a waist thickness of 3 mm. The actual blade dimensions are in the order of tens of centimeters. The sample with film-cooling holes in this study is a scaled-down and simplified version of an actual turbine blade. It can be clamped in a high-temperature furnace for creep testing, facilitating the simulation of the real service environment of turbine blades. The sample dimensions in this study comply with the creep test standard “Standard Test Methods for Conducting Creep, Creep-Rupture, and Stress-Rupture Tests of Metallic Materials” (ASTM E139-2011) published by the American Society for Testing Materials in 2011.

**Figure 2:**
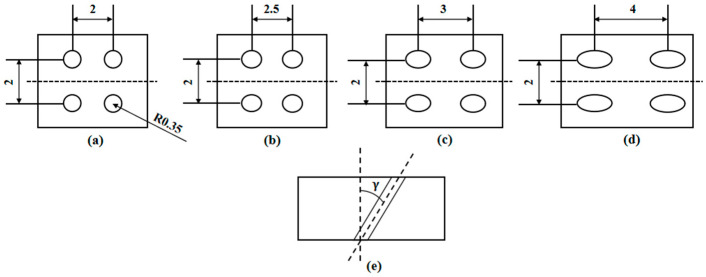
Schematic diagrams of film-cooling holes with different deflection angles: (**a**) 0°; (**b**) 30°; (**c**) 45°; (**d**) 60°; (**e**) diagram of film-cooling-hole inclination angle.

The two-row, four-hole structure shown in Figure 2 is used to simulate the arrangement of film-cooling holes on the blade. To investigate the effect of film-cooling-hole inclination angles on the creep performance of the blade material, specimens with inclination angles of 0°, 30°, 45°, and 60° were designed, as illustrated in the figure. To eliminate the influence of the hole-machining process [23], all film-cooling holes were fabricated using electrical discharge machining (EDM) on the same machine(Manufactured by DEBAINUO, Suzhou, China).

### 2.3. Experimental Method

The creep tests were conducted in accordance with the national standard “Metallic Materials–Creep and Stress-Rupture Test in Tension” (GB/T 2039-2012) published by the Standardization Administration of China in 2012. The test equipment used was an RDL-50 electronic creep rupture testing machine. The test temperature was set to 800 °C, and the nominal stress was set to 350 MPa, based on the yield strength obtained from uniaxial tensile tests of the IN-738 specimens. For each inclination angle (0°, 30°, 45°, and 60°), two flat-plate specimens were tested. To ensure consistency and reliability of the results, all creep tests were performed on the same testing machine under identical conditions. After the completion of the creep tests, the fractured specimens were sectioned, and the fracture morphology around the film hole and the creep fracture surface was analyzed using a scanning electron microscope (Manufactured by ThermoFisher Scientific, Brno, Czech Republic).

## 3. Results

### 3.1. Creep Life and Deformation

**Figure 3:**
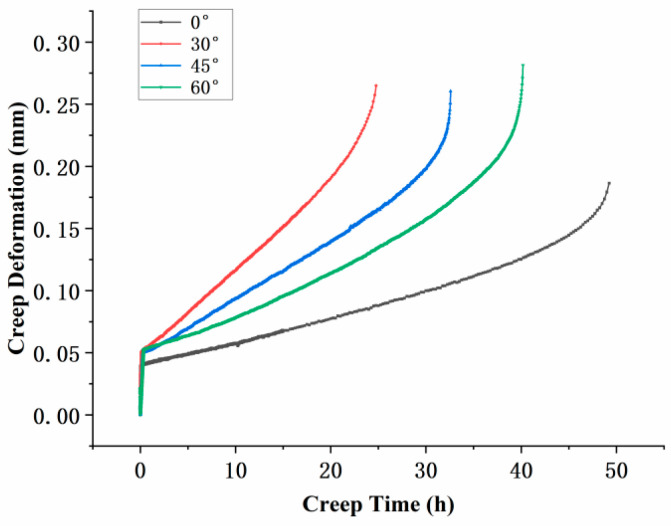
Creep curves of specimens with four groups of angles.

Creep tests were conducted under conditions of 800 °C and 350  MPa, and the results for specimens with different inclination angles are shown in Figure 3. The initial vertical line in the test represents the instantaneous elastic deformation upon completion of stress loading. All specimens exhibited the typical three-stage creep behavior: primary creep with a decreasing creep rate, secondary creep with a nearly constant rate, and tertiary creep with a rapidly increasing rate leading to fracture. The creep lifetimes of the specimens, in descending order, were 0°, 60°, 45°, and 30°. This indicates that the inclination angle of the film-cooling holes has a significant impact on the creep life of the specimens. Moreover, the trend suggests that as the inclination angle increases, the creep life initially decreases and then increases. This observation highlights the complex relationship between the inclination angle and the mechanical performance of the material under high-temperature creep conditions.

From the comparison of post-fracture strain deformation, noticeable differences in creep elongation were observed. The maximum elongation values for the specimens with different film-cooling-hole inclination angles were as follows: 0° specimen, 0.19 mm; 30° specimen, 0.27 mm; 45° specimen, 0.26 mm; and 60° specimen, 0.28 mm. These results indicate that the inclination angle of the film-cooling holes significantly affects the creep deformation of the specimens, with higher inclination angles generally leading to greater creep elongation.

### 3.2. Fracture Morphology Analysis

**Figure 4:**
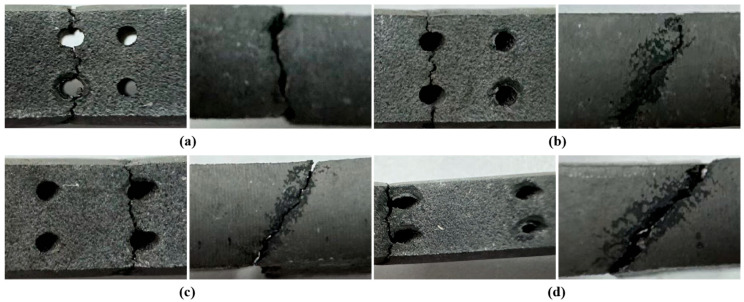
Macroscopic fracture of specimens with four angles: (**a**) 0°; (**b**) 30°; (**c**) 45°; (**d**) 60°.

Figure 4 presents the macroscopic fracture images of specimens with film-cooling holes at four inclination angles after creep rupture. Due to the high-temperature testing environment in air, slight oxidation was observed at the fracture surfaces. As the inclination angle increased, the necking at the fracture site became more pronounced. The fracture surfaces of all specimens indicate that creep failure initiated at the edges of the film-cooling holes and then propagated along the hole. The fracture path remained perpendicular to the loading direction. However, as the inclination angle increased, the crack initiation site shifted progressively from the center of the hole toward the edge. This shift can be attributed to the reduction in the cross-sectional area perpendicular to the loading direction with increasing inclination angle. Consequently, the minimum cross-sectional area (i.e., the critical cross-section) moved from the hole center toward the edge. Side-view observations of the fracture surfaces further revealed that as the inclination angle increased, the angle between the fracture plane and the vertical direction also increased. Notably, the fracture plane consistently aligned with the inclination angle of the film-cooling holes.

**Figure 5:**
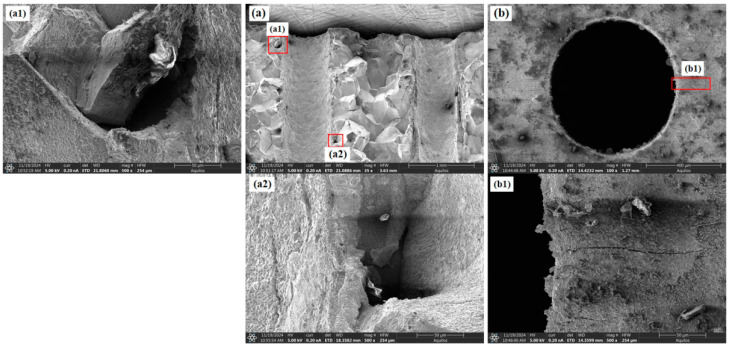
Morphological characteristics of the 0° specimen: (**a**) fracture surface; (**b**) around the hole.

SEM analysis is primarily used to understand the reasons for the differences in creep behavior among specimens with different inclination angles from a microscopic perspective. Figure 7a and Figure 8a both show the fracture surface morphology, but from different viewing angles, and Figures such as a1, a2, and b1 show magnified details. Figure 5 shows the fracture morphology and features around the holes for the 0° film-cooling-hole specimen after creep rupture. The fracture surface exhibited significant roughness with pronounced dimpled (wavy) characteristics. These dimpled regions were formed by the aggregation of microcracks and micropores, resulting in a rough fracture surface. The interior of the film-cooling holes appeared relatively smooth, with a few small voids visible at the edges of the ruptured holes. The surface around the unbroken holes remained smooth and intact, with only a few fine cracks observed at the edges. These cracks consistently propagated perpendicular to the loading direction. The overall fracture surface displayed minimal undulations along the loading direction and remained relatively smooth.

**Figure 6:**
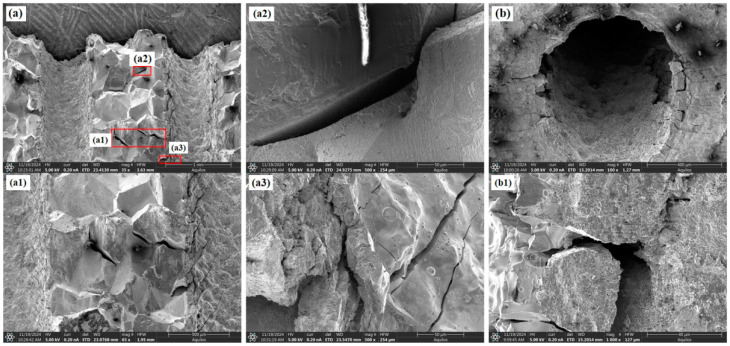
Morphological characteristics of the 30° specimen: (**a**) fracture surface; (**b**) around the hole.

**Figure 7:**
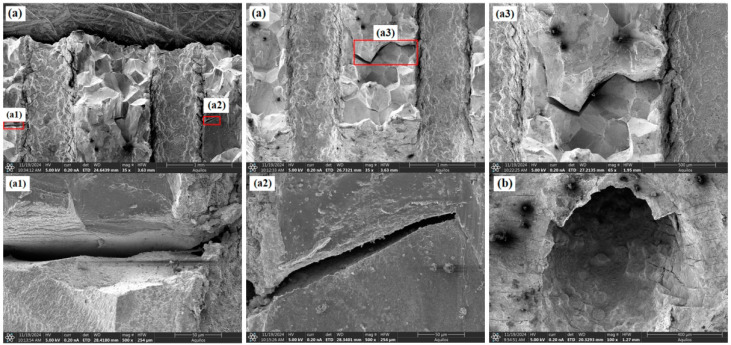
Morphological characteristics of the 45° specimen: (**a**) fracture surface; (**b**) around the hole.

**Figure 8:**
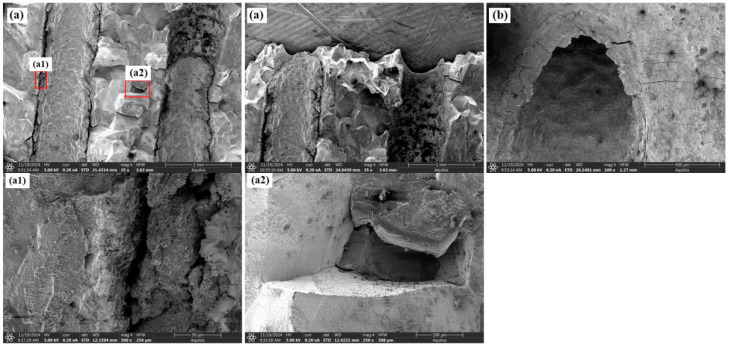
Morphological characteristics of the 60° specimen: (**a**) fracture surface; (**b**) around the hole.

As shown in Figure 6 and Figure 7, the fracture surfaces of the 30° and 45° specimens also exhibited dimple-type rupture. However, the number of voids and cracks between adjacent holes and at the hole edges significantly increased compared to the 0° specimen. The increased inclination of the film-cooling holes resulted in more severe stress concentration at the hole edges, causing cracks to initiate at the edges and propagate outward. Cracks between adjacent holes almost penetrated the middle region. For the 60° specimen (as shown in Figure 8), the number of cracks and voids was lower than those in the 30° and 45° specimens.

In summary, the creep rupture of specimens with film-cooling holes was primarily caused by stress concentration at the hole edges, leading to dimple-type fracture. SEM analysis, differences in defect voids, and cracks around the hole and near the fracture surface were observed among specimens with different inclination angles. It is inferred that the specimen’s lifespan is positively correlated with the number of cracks and voids around the hole, which microscopically explains the variation in creep life.

## 4. Numerical Analysis

### 4.1. Creep Damage Constitutive Model

The K-R damage model was used for finite element simulations of the specimens. According to the K-R damage model, the degree of material damage is measured by the damage factor *D*. Physically, *D* represents the volume fraction of microcracks and voids within the material. Specifically: (1) *D* = 0: The material is undamaged. (2) 0 < *D* < 1: The material is in a damaged state. (3) *D* = 1: The material undergoes catastrophic failure. In practical situations, when the damage factor reaches a critical value near 1, the material rapidly deteriorates and ultimately fails. This value is referred to as the material’s critical damage factor. Assuming the initial cross-sectional area of the material is $S_{0}$, the effective area is $S_{0}$(1 − *D*), and the corresponding net stress can be expressed as follows:

(1) $$\sigma^{*}={\frac{\sigma }{1-D}}^{*}$$

The constitutive relationship of the creep process is as follows:

(2) $${\dot{\varepsilon }}_{c}=A\frac{\sigma_{eq}^{n}}{{\left(1-D\right)}^{n}}\omega$$

(3) $$\dot{D}=B\frac{\sigma_{eq}^{k}}{{\left(1-D\right)}^{\varphi }}$$

**Figure 9:**
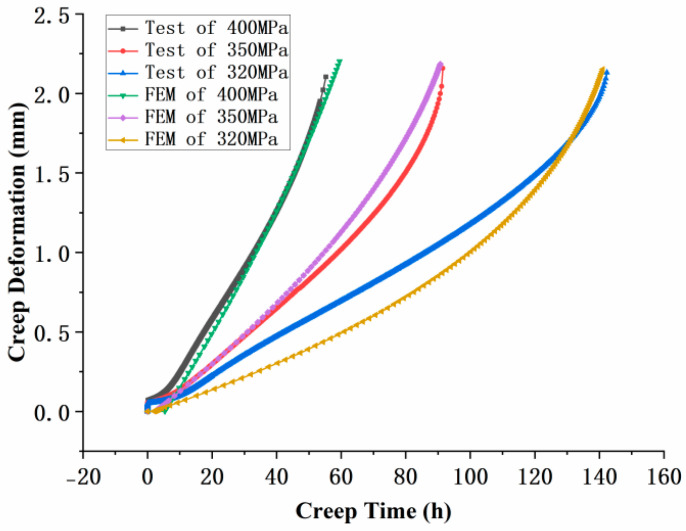
Experimental results under three stress conditions and parameter simulation validation.

In the equation, ${\dot{\varepsilon }}_{c}$ and $\dot{D}$ represent the creep rate and damage rate, respectively, and $\sigma_{eq}$ is the equivalent stress. *A*, *B*, *n*, *k*, and φ are material constants that can be determined from the material’s creep curve. As shown in Figure 9, creep tests were performed on cylindrical specimens at 800 °C under three different stress levels (320 MPa, 350 MPa, and 400 MPa). The parameters of the K-R model were fitted based on the test results. The fitted K-R model parameters are listed in Table 2.

Figure 9 shows the validation results of the model, demonstrating good agreement between the model parameters and the experimental creep test data.

### 4.2. Finite Element Simulation

**Figure 10:**
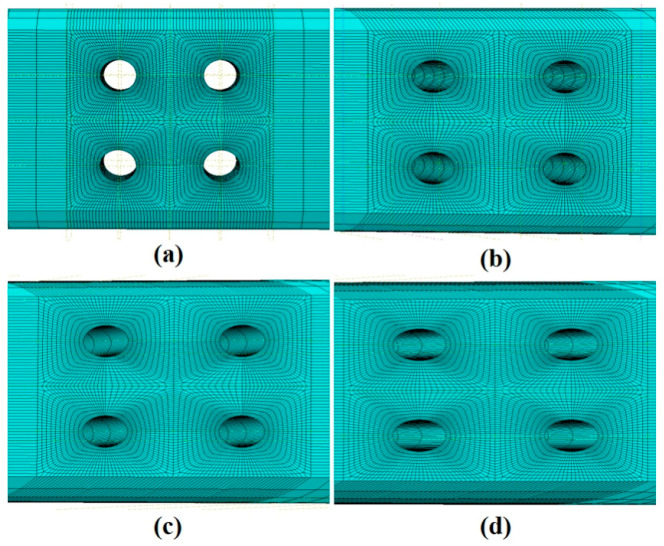
Mesh division diagrams of film-cooling holes with four angles: (**a**) 0°; (**b**) 30°; (**c**) 45°; (**d**) 60°.

**Figure 11:**
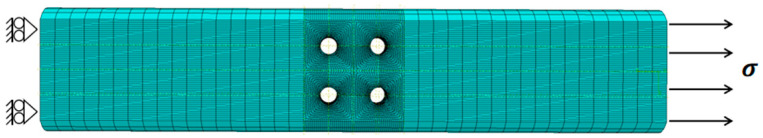
Boundary condition schematic diagram.

**Figure 12:**
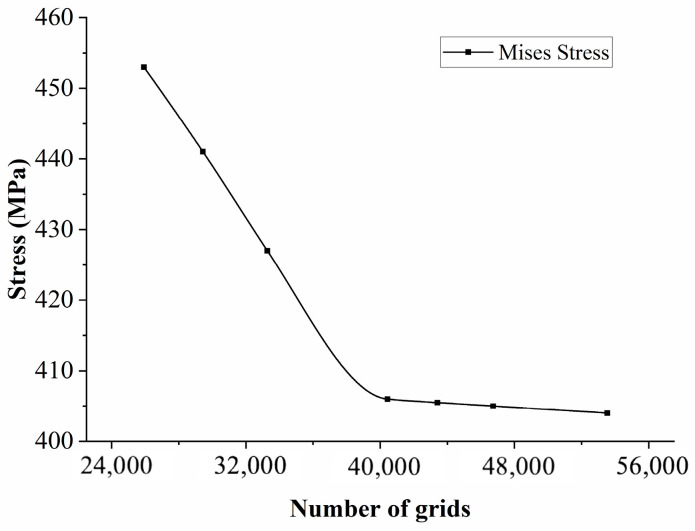
Mesh independence verification.

The constitutive model described above was implemented into Abaqus using a CREEP subroutine. Finite element models of the gauge sections for the specimens with four different film-cooling-hole inclination angles were established. The mesh was generated using C3D8 hexahedral elements, as shown in Figure 10, with mesh refinement applied around the edges of the film-cooling holes. Apply a fixed constraint at one end of the model and a uniformly distributed load of 350 MPa at the other end, as shown in Figure 11. To ensure the accuracy of the finite element simulation results, a mesh independence verification was conducted near the film-cooling hole, as shown in Figure 12. It can be observed that when the number of elements exceeds 40,438, the stress values gradually converge. The simulation was terminated when the damage factor approached a value of 1.

### 4.3. Results Analysis

#### 4.3.1. Strain Analysis

**Figure 13:**
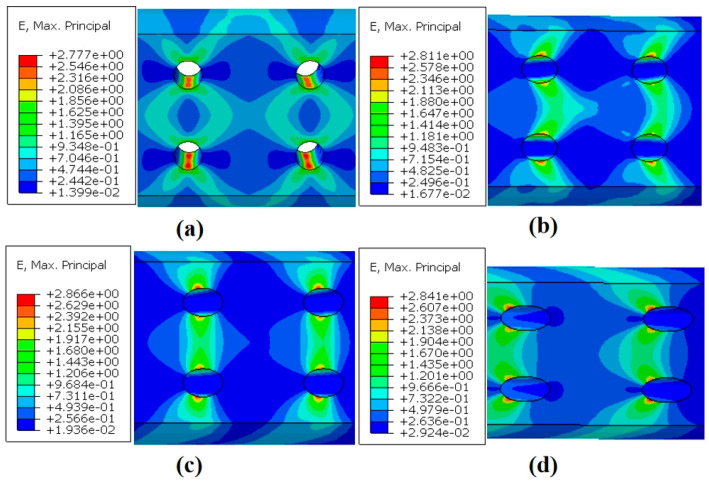
Strain distribution at the maximum principal strain node of specimens with four angles: (**a**) 0°; (**b**) 30°; (**c**) 45°; (**d**) 60°.

Figure 13a–d present the strain contours for specimens with four different inclination angles at the end of the simulation. The results indicate that the creep strain between the two longitudinally aligned film-cooling holes is greater than the strain between the two holes in the same transverse row. Creep deformation is significantly concentrated around the film-cooling holes, with much smaller deformation in regions far from the holes. As the inclination angle of the film-cooling holes increases, the path of maximum strain between the two longitudinal holes, as well as the location of the maximum stress on the hole edges, gradually shifts toward the left end. This can be attributed to the fact that the minimum cross-sectional area perpendicular to the loading direction (i.e., the critical section) shifts from the center of the holes toward the left edge as the inclination angle increases.

**Figure 14:**
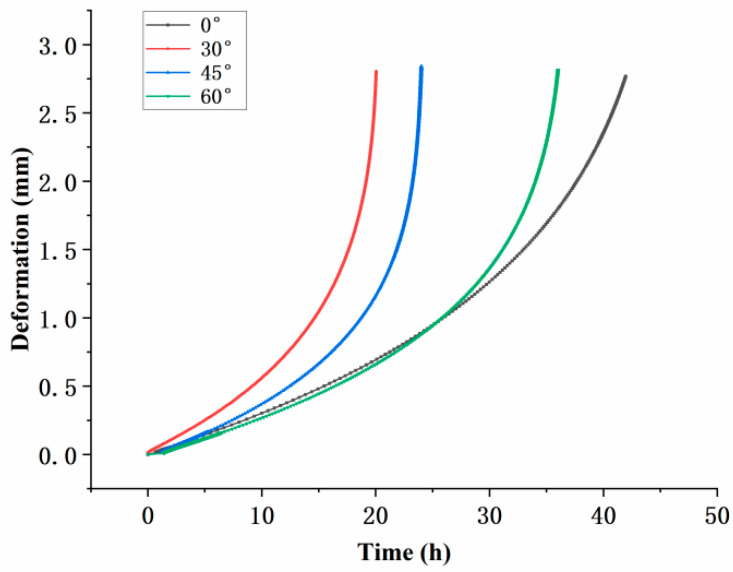
Variation in strain at the maximum principal strain node with time.

The variation in the maximum principal strain at the critical node with creep time for the four inclination angles is shown in Figure 14. The 30° film-cooling hole specimen exhibited the fastest strain growth, with a continuously increasing creep rate, leading to the earliest fracture and indicating the shortest creep life. In contrast, the 0° film-cooling hole specimen had the longest steady-state creep phase. The overall trends of the strain curves for specimens with different inclination angles are consistent with the results of the creep experiments.

#### 4.3.2. Stress Analysis

**Figure 15:**
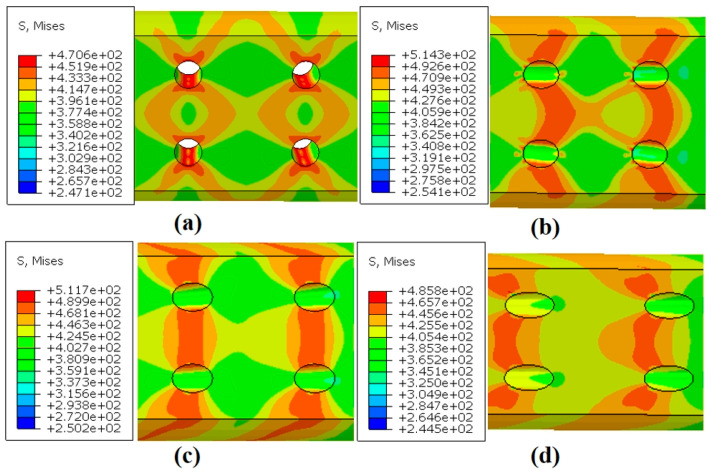
Mises stress distribution of specimens with four angles: (**a**) 0°; (**b**) 30°; (**c**) 45°; (**d**) 60°.

Figure 15a–d present the maximum Mises stress contours for the four inclination angles at the end of the simulation. All specimens exhibited varying degrees of stress concentration around the film-cooling holes. The shape of the stress concentration region changed significantly with the inclination angle. For the 0° holes, the stress distribution around the hole edges was the most uniform, showing a butterfly-shaped divergent pattern. As the inclination angle increased, the high-stress regions gradually shifted toward the left edge of the holes.

**Figure 16:**
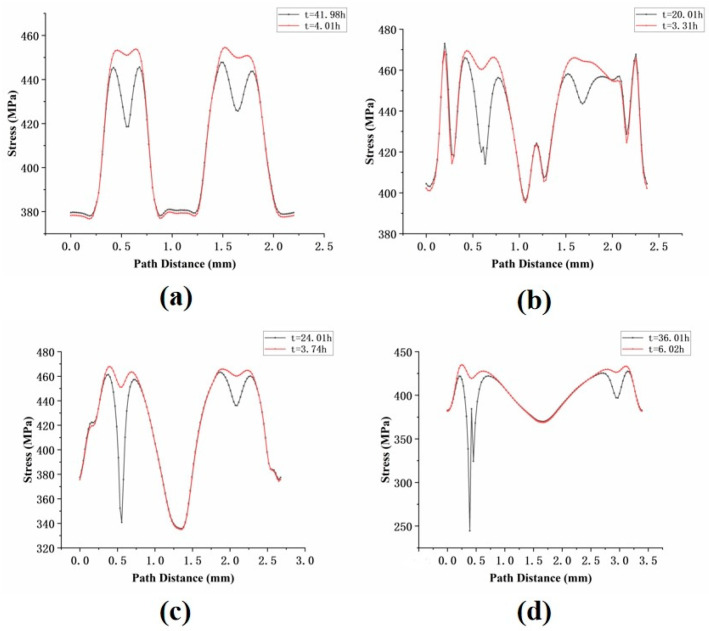
Stress distribution around the holes of specimens with four angles: (**a**) 0°; (**b**) 30°; (**c**) 45°; (**d**) 60°.

As shown in Figure 16, the stress distribution around the holes for the four inclination angles is presented. The results indicate that the stress around the holes during the initial stage of creep was generally higher than in the later stages, suggesting that stress redistribution occurred during the creep process. Although the degree of stress concentration decreased with creep time, the stress values for the 30° and 45° specimens remained consistently higher than those for the other two specimens. This observation aligns with their shorter creep life observed in the experimental results.

#### 4.3.3. Creep Damage Analysis

**Figure 17:**
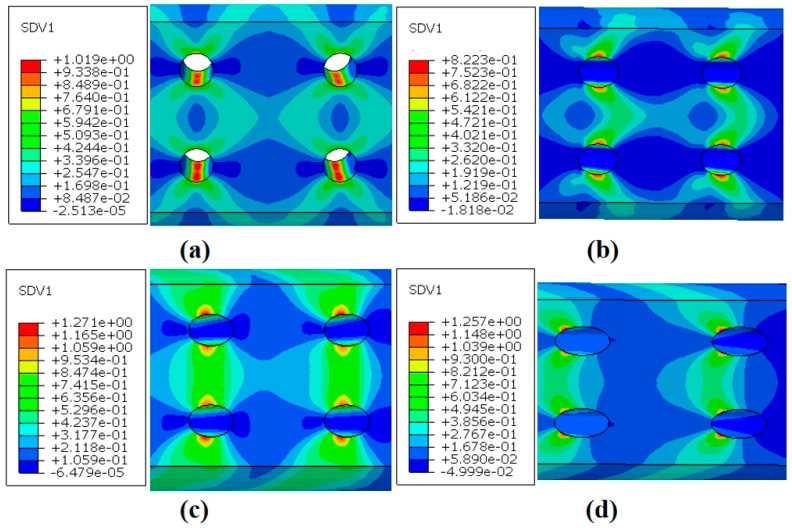
Creep damage distribution of specimens with four angles: (**a**) 0°; (**b**) 30°; (**c**) 45°; (**d**) 60°.

Figure 17a–d present the total creep damage contours for the four inclination angles at the end of the simulation. The results show that regions far from the film-cooling holes exhibited almost no damage, while the areas near the holes experienced significant stress concentration, resulting in the maximum creep damage. There was a substantial difference in damage between the regions near the film-cooling holes and those farther away, indicating that the damage and fractures were initiated in areas with higher stress. The distribution of the maximum damage path aligned closely with the paths of maximum stress and strain.

**Figure 18:**
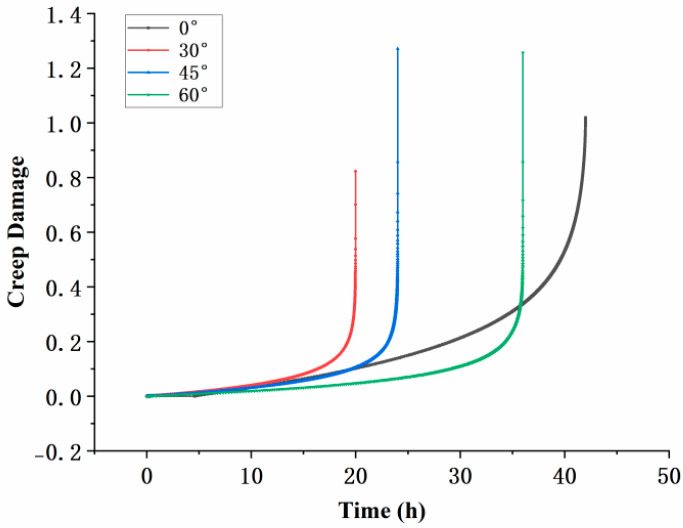
Variation in creep damage with time.

The variation in the maximum creep damage value with time for specimens at different inclination angles is presented in Figure 18. During the creep process, the creep damage for the 30° film-cooling-hole specimen remained consistently higher than that of the other three inclination angles. It reached the critical value and failed the fastest, resulting in the shortest creep life, which is consistent with the experimental results. Moreover, the simulation for the 30° specimen terminated prematurely before the creep damage reached 1. This may be attributed to the high stress at the hole edges, causing convergence issues in the calculation.

#### 4.3.4. Simulation Path Analysis

**Figure 19:**
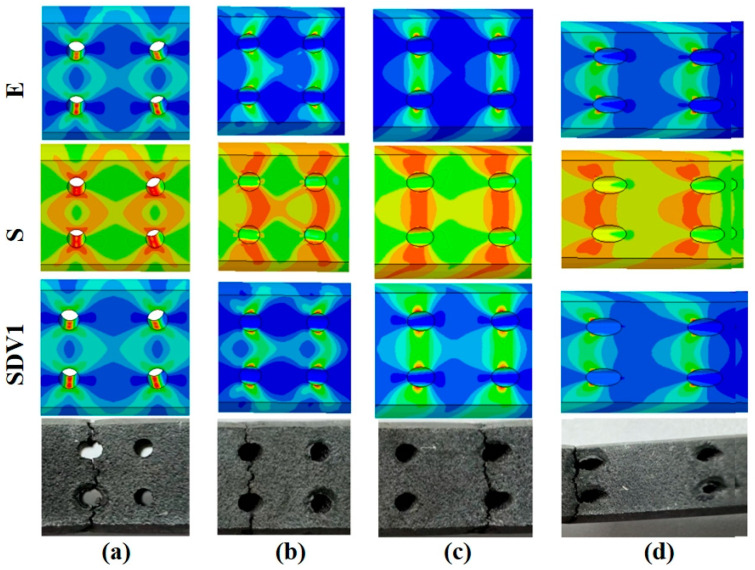
Comparison between simulation distribution cloud diagrams and fracture paths of specimens with four angles: (**a**) 0°; (**b**) 30°; (**c**) 45°; (**d**) 60°.

As shown in Figure 19, the strain, stress, and damage distribution contours for the four inclination angles are compared with the experimental fracture paths of the specimens. For the inclined holes (30°, 45°, and 60°), the simulated paths of maximum strain, stress, and damage align closely with the experimentally observed fracture paths. For the 0° specimen, the simulation contours show a butterfly-shaped divergent distribution of maximum strain, stress, and damage. The distribution is symmetric in both horizontal and vertical directions. Due to the combined effects of symmetry, the cracks ultimately propagated in a direction perpendicular to the stress-loading axis. This conclusion is supported by the stress distribution along the direction perpendicular to the stress-loading axis inside the holes.

## 5. Discussion

Creep life is a crucial indicator for evaluating creep performance. This section focuses on the prediction of creep life for specimens with film hole structures. In engineering applications, film hole structures are typically subjected to multiaxial stress, making them representative of a multiaxial stress state. However, existing life prediction methods remain inefficient and inconvenient [24]. Studies have shown that combining the continuum damage model with the nodal stress method can accurately predict creep life under multiaxial stress conditions [25]. Establishing the relationship between geometric parameters and stress states through numerical simulation helps simplify and improve the accuracy of creep rupture life prediction under multiaxial stress.

Numerical calculations and relevant literature indicate that under symmetric geometries and simple loading conditions, there exists a specific node in the structure where the stress remains nearly constant and is unaffected by material creep properties, such as the stress exponent *n* [26,27]. Therefore, the position of this node can be determined using the simple Norton creep model, as expressed below:

(4) $$\dot{\varepsilon }=A\sigma^{n}$$

First, the creep rate $\dot{\varepsilon }$ is set to be $10^{-5}$. The stress exponent n in the Norton model is assigned values, such as *n* = 1, 3, 5, 7, 10, and the corresponding A values can be calculated from the equation. Different values of A and n represent various creep properties of the material.

**Figure 20:**
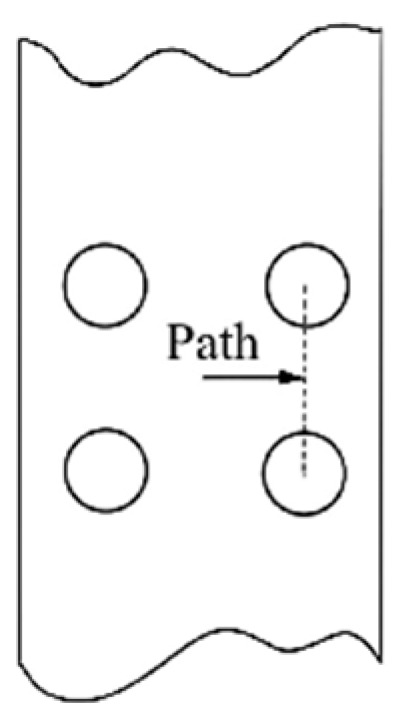
Defined path.

The creep constitutive model was simplified to the Norton model. A path in the multi-hole model, as shown in Figure 20, was selected, which is perpendicular to the stress loading direction. The starting point of the path was defined as the center point between the four holes, and the distance from the starting point to the node was defined as $r^{*}$. The finite element analysis was performed on the multi-hole model to obtain the stress distribution along the defined path.

**Figure 21:**
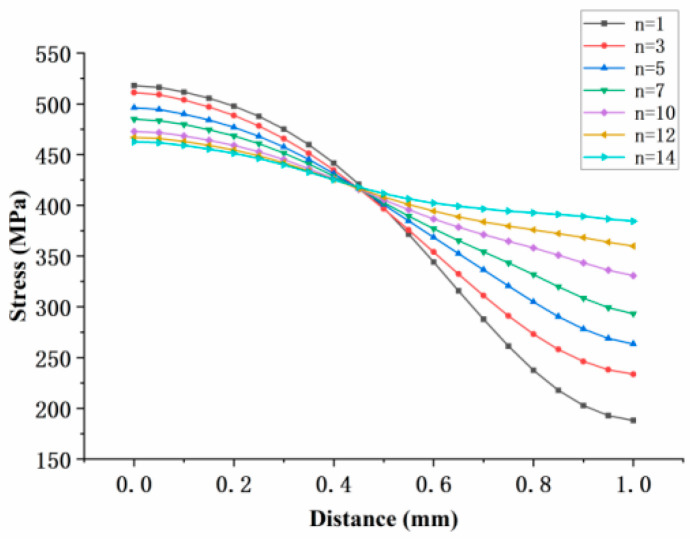
Stress distribution along the path of the 0° model.

The results, as shown in Figure 21, indicate that the Mises stress distributions along the defined path for the 0° straight-hole model under different Norton parameters intersect at a specific node. The intersection point was determined to be at a distance of $r^{*}$ = 0.45 mm, with a stress value of σ = 415 MPa.

**Figure 22:**
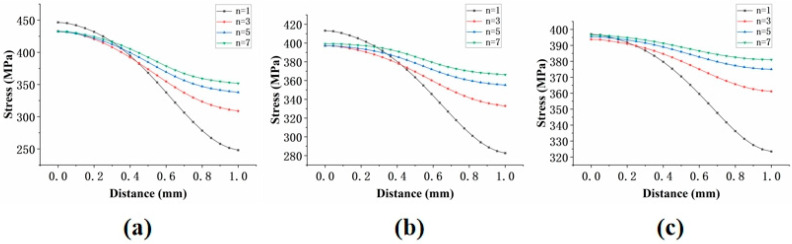
Stress distribution of specimens with three angles under different *n*-values: (**a**) 30°; (**b**) 45°; (**c**) 60°.

Further research revealed that when the film-cooling holes have an inclination angle, the stress distribution curves for different Norton stress exponents (*n*) no longer intersect, as the node location is searched by varying n, as shown in Figure 22. The analysis suggests that the inclined film-cooling holes disrupt the original symmetry present in the straight-hole model, resulting in the disappearance of the intersection node.

**Figure 23:**
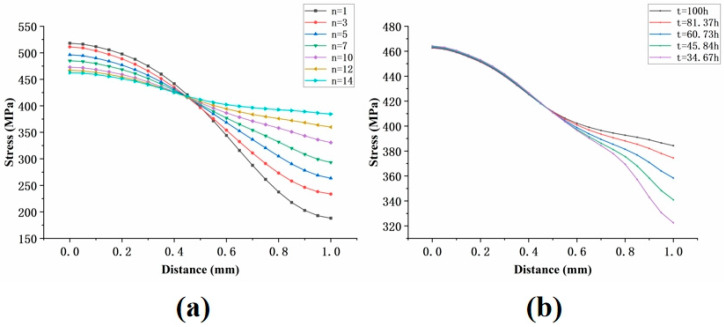
Two methods for determining the node location: (**a**) different n-values; (**b**) different time points.

A subsequent literature review [19] identified two main methods for determining the node location: (1) varying the Norton stress exponent n to identify the intersection point, and (2) analyzing the stress distribution at different time points to locate the point where the stress state remains constant over time. To verify the consistency between these two methods, the results were compared, as shown in Figure 23.

**Figure 24:**
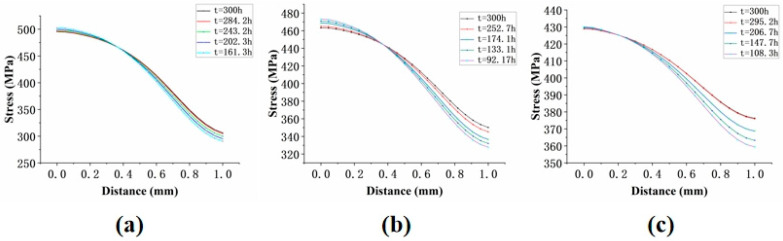
Stress distribution of specimens with three angles at different time points: (**a**) 30°; (**b**) 45°; (**c**) 60°.

It can be concluded that the results of the two methods for determining the node location are consistent. Therefore, for inclined holes, the node location can be identified by analyzing the stress distribution along the path at different time points. The results are shown in Figure 24.

Taking the initial time $t_{0}$ = 0 and initial damage $D_{0}$ = 0, integrating Equation (3) over the range *D* = 0 to *D* = 1, the creep life can be expressed as follows:

(5) $$t_{r}=\frac{1}{B(1+\varphi )\sigma^{k}}$$

The parameters in the formula were obtained from Table 2 in Section 3.1. By substituting the value of φ,k,B and the node stress σ, the predicted creep life values were calculated, as shown in Table 3. The prediction error for both the smooth specimens and the specimens with different inclination angles was within 15%.

**Figure 25:**
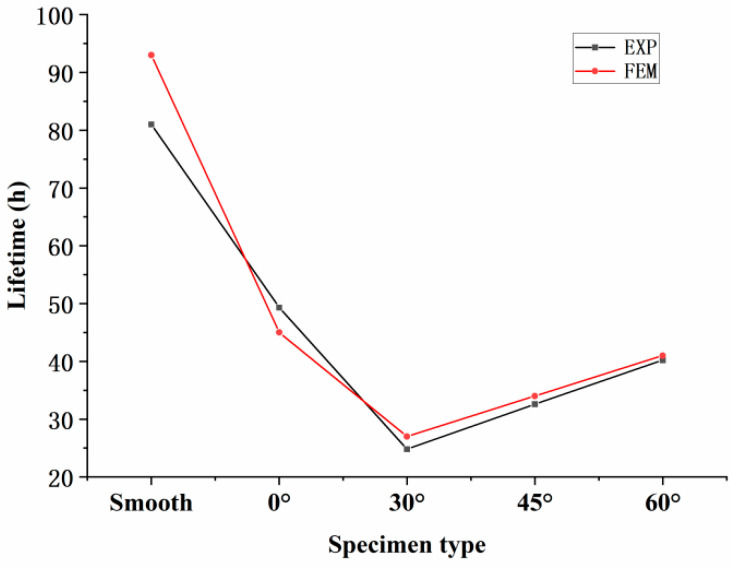
Comparison between test life and predicted life.

The comparison between the experimental and predicted creep lifetimes for different specimens is shown in Figure 25. The predicted lifetime exhibits a trend of first decreasing and then increasing within the inclination angle range of 0° to 60°. This trend is consistent with the experimental lifetime results.

The creep life prediction process can be summarized as follows:

1. Obtain material parameters through uniaxial creep tests.
2. Use finite element simulations to determine the node location and obtain the stress value at the node.
3. Calculate the creep life using the Formula (5) and compare the results with experimental data for validation.

## 6. Conclusions

In this study, high-temperature creep tests were conducted on IN-738 alloy specimens with different film-cooling-hole inclination angles under conditions of 800 °C and 350 MPa to investigate the effect of hole inclination on the creep performance of nickel-based superalloy IN-738. The findings provide a reference for the design of film-cooling-hole angles in actual turbine blades to achieve better structural strength and lifespan characteristics. Future research could explore the effect of inclination angles on fatigue performance or investigate the influence of film-cooling holes with different geometric shapes on the creep behavior of the specimens. The conclusions are as follows:

1. Under identical testing conditions, the inclination angle of film-cooling holes has a significant impact on the creep performance of the specimens. The creep life decreases in the order of 0° > 60° > 45° > 30°. SEM observations of the fracture surfaces revealed that the creep fracture in specimens with film-cooling holes is primarily caused by stress concentration at the hole edges, with cracks typically initiating at these locations. The creep fracture mode is a dimple-type failure, which occurs in regions of stress concentration around the film-cooling holes.
2. Numerical analyses of specimens with different inclination angles showed that the presence of film-cooling holes disrupts the geometric continuity of the specimens, leading to localized concentrations of strain, stress, and damage around the holes. The simulated fracture paths were highly consistent with the actual crack paths observed in the specimens.
3. The node stress method was used to predict the creep rupture life of specimens with different film-cooling-hole inclination angles. The predicted results agreed well with the experimental data, demonstrating that the node stress method provides high accuracy for creep life prediction.

## Figures and Tables

**Table 1:** Chemical compositions of nickel-based superalloy IN-738.

Element	wt (%)	Element	wt (%)
C	0.16	Al	3.3
Si	0.22	Fe	0.3
Mn	0.15	Mo	1.7
W	2.45	Ti	3.4
S	0.005	Zr	0.07
Cr	15.93	Ta	1.6
Ni	Balance	Nb	0.8
Co	8.3	B	0.008

**Table 2:** Parameters of the K-R damage model.

*A*	*B*	*n*	*k*	***φ***
$1.81\times 10^{-14}$	$6.43\times 10^{-15}$	4.72	4.28	20.61

**Table 3:** Predicted life and error.

Specimen	Node Stress	Test Life	Predicted Life	Error
Smooth	350 MPa	81 h	93 h	14.8%
0°	415 MPa	49.3 h	45 h	8.7%
30°	466 MPa	24.8 h	27 h	8.9%
45°	444 MPa	32.6 h	34 h	4.3%
60°	425 MPa	40.2 h	41 h	2.0%

## Data Availability

The original contributions presented in this study are included in the article. Further inquiries can be directed to the corresponding author.
